# Supplementary material for: Status and associated factors of psychological resilience of Chinese medical aid team members under public health emergencies in a cross-sectional study
Source: PLoS One. 2025 Jun 17;20(6):e0324952. doi: 10.1371/journal.pone.0324952 (PMC12173397; doi:10.1371/journal.pone.0324952)
Supplement: Attachment — Submitted filename: Response to Reviewers.docx [file pone.0324952.s003.docx]

**PONE-D-25-03434**

**Status and Associated Factors of Psychological Resilience of Chinese Medical Aid Team Members under Public Health Emergencies: A Cross-Sectional Study**

**PLOS ONE**

Dear Editor-in-Chief,

Thank you very much for your careful review and constructive suggestions with regard to our manuscript **“Status and Associated Factors of Psychological Resilience of Chinese Medical Aid Team Members under Public Health Emergencies: A Cross-Sectional Study”.**

We have carefully evaluated all the comments and suggestions, and responded to them in our revised manuscript accordingly. All the authors agreed upon and approved the contents of the manuscript. Again, we appreciate the scholarly work by the Editors/Reviewers, and hope our detailed revisions have addressed all the criticisms raised by the reviewers.

Please contact us with any further questions and we are looking forward to your positive consideration.

Sincerely,

Yinhua Liang

Teaching and Research Section of Clinical Nursing

Xiangya Hospital

Central South University
Changsha, 410008, China

liangyh@csu.edu.cn
Please include the following items when submitting your revised manuscript:

- A rebuttal letter that responds to each point raised by the academic editor and reviewer(s). You should upload this letter as a separate file labeled 'Response to Reviewers'.

**Answer:** Thank you for your suggestion.

We have included a separate **"Response to Reviewers"** document addressing each point raised.

- A marked-up copy of your manuscript that highlights changes made to the original version. You should upload this as a separate file labeled 'Revised Manuscript with Track Changes'.

**Answer:** Thank you for your reminder.

We have provided a marked-up copy of the manuscript labeled **'Revised Manuscript with Track Changes'** for your review.

- An unmarked version of your revised paper without tracked changes. You should upload this as a separate file labeled 'Manuscript'.

**Answer:** Thank you for your reminder.

We have provided an unmarked version of the revised paper labeled **'Manuscript'** for your review.

If you would like to make changes to your financial disclosure, please include your updated statement in your cover letter. Guidelines for resubmitting your figure files are available below the reviewer comments at the end of this letter.
**Answer:** Thank you for your notice.

We do not need to make any changes to our financial disclosure. In addition, we have followed the guidelines provided for resubmitting the figure files.

If applicable, we recommend that you deposit your laboratory protocols in protocols.io to enhance the reproducibility of your results. Protocols.io assigns your protocol its own identifier (DOI) so that it can be cited independently in the future. For instructions see: [https://journals.plos.org/plosone/s/submission-guidelines#loc-laboratory-protocols](https://track.editorialmanager.com/CL0/https:%2F%2Fjournals.plos.org%2Fplosone%2Fs%2Fsubmission-guidelines%23loc-laboratory-protocols/1/010f01955b273b4b-cb50b722-1bb9-4e12-930b-64ec03c1f07d-000000/-SvK5TS3gtro4aQEQe13IbKxpsG3QqFd4q-ft-WxUwM=200). Additionally, PLOS ONE offers an option for publishing peer-reviewed Lab Protocol articles, which describe protocols hosted on protocols.io. Read more information on sharing protocols at [https://plos.org/protocols?utm_medium=editorial-email&utm_source=authorletters&utm_campaign=protocols](https://track.editorialmanager.com/CL0/https:%2F%2Fplos.org%2Fprotocols%3Futm_medium=editorial-email%26utm_source=authorletters%26utm_campaign=protocols/1/010f01955b273b4b-cb50b722-1bb9-4e12-930b-64ec03c1f07d-000000/4fWhURDzuJuvSzUmMdgTRijlxjMj8Wg6R_NKwkeCW5M=200).
**Answer:** Thank you for the thoughtful suggestion.

Our team carefully considered your recommendation. However, as we have provided a detailed description of our methods in the manuscript, we are not planning to deposit our laboratory protocols in protocols.io at this time. Nonetheless, we sincerely appreciate the suggestion and will keep it in mind for future projects.

Journal requirements:

When submitting your revision, we need you to address these additional requirements.

1. Please ensure that your manuscript meets PLOS ONE's style requirements, including those for file naming. The PLOS ONE style templates can be found at

https://journals.plos.org/plosone/s/file?id=wjVg/PLOSOne_formatting_sample_main_body.pdf and

[https://journals.plos.org/plosone/s/file?id=ba62/PLOSOne_formatting_sample_title_authors_affiliations.pdf](https://track.editorialmanager.com/CL0/https:%2F%2Fjournals.plos.org%2Fplosone%2Fs%2Ffile%3Fid=ba62%2FPLOSOne_formatting_sample_title_authors_affiliations.pdf/1/010f01955b273b4b-cb50b722-1bb9-4e12-930b-64ec03c1f07d-000000/gaXIujPqLsJjQSFrb2fWSp-CgGdKcIXmPZ_EfQTx08M=200).

**Answer:** Thank you for the suggestion.

We have carefully reviewed the PLOS ONE's style requirements and ensured that both our manuscript and file names fully comply with the provided guidelines.

2. Please provide additional details regarding participant consent. In the ethics statement in the Methods and online submission information, please ensure that you have specified (1) whether consent was informed and (2) what type you obtained (for instance, written or verbal, and if verbal, how it was documented and witnessed). If your study included minors, state whether you obtained consent from parents or guardians. If the need for consent was waived by the ethics committee, please include this information.

If you are reporting a retrospective study of medical records or archived samples, please ensure that you have discussed whether all data were fully anonymized before you accessed them and/or whether the IRB or ethics committee waived the requirement for informed consent. If patients provided informed written consent to have data from their medical records used in research, please include this information.

**Answer:** Thank you for the suggestion.

In section 2.5 Ethical considerations, we showed that the study was approved by the Ethics Committee of Xiangya Hospital, Central South University (registry number 2025010116). The study complied with the Declaration of Helsinki and all participants signed informed consent forms and their participation was anonymous. We have also uploaded the attachment of the corresponding ethical approval certificate.

3.  We note that the grant information you provided in the ‘Funding Information’ and ‘Financial Disclosure’ sections do not match.

When you resubmit, please ensure that you provide the correct grant numbers for the awards you received for your study in the ‘Funding Information’ section.

**Answer:** Thank you for your reminder.

We have ensured that we provide the correct grant numbers for the awards in the ‘Funding Information’ section.

This work was funded by the National Natural Science Foundation of China [82160554], Natural Science Foundation of Hunan Province [2020JJ8051]. And supported by the Project Program of National Clinical Research Center for Geriatric Disorders [2021LNJJ15].

4.  Thank you for stating the following financial disclosure:

 [This work was funded by the National Natural Science Foundation of China [82160554], Natural Science Foundation of Hunan Province [2020JJ8051]. And supported by the Project Program of National Clinical Research Center for Geriatric Disorders [Xiangya Hospital, Grant No. 2021LNJJ15].].

Please state what role the funders took in the study.  If the funders had no role, please state: ""The funders had no role in study design, data collection and analysis, decision to publish, or preparation of the manuscript.""

**Answer:** Thank you for your reminder.

We have clarified in the cover letter that the funders provided support for the material printing cost during the questionnaire collection and the layout cost during the later publication of the article but had no role in study design, data analysis, decision to publish, or preparation of the manuscript.

If this statement is not correct you must amend it as needed.

Please include this amended Role of Funder statement in your cover letter; we will change the online submission form on your behalf.

**Answer:** Thank you for the suggestion.

The statement is correct.

Additional Editor Comments:

Dear Authors,

Thank you for your submission to PLOS ONE. The reviewers have provided constructive feedback to enhance the clarity, rigor, and impact of your manuscript. While your study presents valuable insights into preoperative readiness among patients undergoing arteriovenous fistula percutaneous transluminal angioplasty, several areas require revision to strengthen the manuscript's coherence, methodological transparency, and discussion depth.
**Answer:** Thank you very much for considering our work and for your valuable guidance.

We have revised our manuscript one by one according to all reviewers' comments and provided a peer-to-peer response. Additionally, we have made all modifications in blue color. We are looking forward to your positive consideration.

Reviewer 1 highlights the need for aligning the manuscript with journal guidelines, particularly in reporting methodological details, defining the research problem, and emphasizing the study’s practical applications. Additionally, more comparisons with previous studies in the discussion section, along with citations from suggested references, will improve the scholarly context of your work.

**Answer:** Thank you for your valuable suggestion.

According to reviewer 1's comments, we have revised the methodological part of the manuscript according to the journal guidelines, clarified the research problem, emphasized practical applications, and compared previous studies in the discussion section, along with citations from suggested references.

Reviewer 2 suggests refining the abstract by specifying the study duration and clarifying its implications. The introduction would benefit from a smoother transition into the psychological aspects of preoperative readiness, and more recent references should be included. The methodology section requires additional details on participant selection, sample size justification, scale reliability, and the rationale for statistical choices. In the results, key findings should be highlighted more explicitly, and assumptions for multivariate analysis should be addressed. The discussion needs a deeper exploration of the correlation between psychological resilience and preoperative readiness, along with more actionable recommendations for practice. Finally, the conclusion should present specific strategies for healthcare implementation, and minor grammatical improvements should be made throughout.

**Answer:** Thank you for your valuable suggestion.

According to reviewer 2's comments, we have revised the abstract, introduction, method, result and discussion.

To proceed, please revise your manuscript accordingly, ensuring that:

The abstract clearly specifies the study period and includes practical implications.

**Answer:** Thank you for your valuable suggestion.

According to reviewer 2's comments, we have revised the abstract.

The introduction provides a well-defined research problem, the significance of the study variables, and a clear rationale for conducting the study.

**Answer:** Thank you for your valuable suggestion.

According to reviewer 2's comments, we have revised the introduction.

The methods section includes details on sampling methods, scale reliability, validation procedures, and justification for statistical analyses.

**Answer:** Thank you for your valuable suggestion.

According to reviewer 2's comments, we have revised the methods section.

The results section presents findings clearly with sufficient interpretation and proper statistical reporting, including effect sizes.

**Answer:** Thank you for your valuable suggestion.

According to reviewer 2's comments, we have revised the results.

The discussion integrates more comparative studies, explores psychological resilience more deeply, and includes targeted intervention strategies.

**Answer:** Thank you for your valuable suggestion.

According to reviewer 2's comments, we have revised the discussion.

The conclusion offers specific recommendations for future research and policy implications.
The language and formatting are refined to improve clarity and precision.

**Answer:** Thank you for your valuable conclusion.

According to reviewer 2's comments, we have revised the abstract.

We encourage you to carefully address the reviewers' concerns and incorporate their suggested references to strengthen your study’s academic contribution. Once revised, please submit a detailed response letter explaining how each point was addressed.
**Answer:** Thank you for your reminder.

We have included references suggested by reviewers to enhance the academic contribution of our research. Additionally, we have included a separate **"Response to Reviewers"** document addressing each point raised.

We look forward to receiving your revised manuscript.

Best regards,

According to reviewer 2's comments, we have revised the abstract, introduction, method, result and discussion.

[Note: HTML markup is below. Please do not edit.]

Reviewers' comments:

Reviewer's Responses to Questions

**Comments to the Author**

1. Is the manuscript technically sound, and do the data support the conclusions?

The manuscript must describe a technically sound piece of scientific research with data that supports the conclusions. Experiments must have been conducted rigorously, with appropriate controls, replication, and sample sizes. The conclusions must be drawn appropriately based on the data presented.

Reviewer #1: Yes

**Answer:** Thank you for your positive feedback.

Reviewer #2: Yes

**Answer:** Thank you for your positive feedback.

2. Has the statistical analysis been performed appropriately and rigorously?

Reviewer #1: Yes

**Answer:** Thank you for your positive feedback.

Reviewer #2: Yes

**Answer:** Thank you for your positive feedback.

3. Have the authors made all data underlying the findings in their manuscript fully available?

The [PLOS Data policy](http://track.editorialmanager.com/CL0/http:%2F%2Fwww.plosone.org%2Fstatic%2Fpolicies.action%23sharing/1/010f01955b273b4b-cb50b722-1bb9-4e12-930b-64ec03c1f07d-000000/-wpNvfQywJk4a0kmxMVSYsA87uvyIBIjf4qrsLscdXc=200) requires authors to make all data underlying the findings described in their manuscript fully available without restriction, with rare exception (please refer to the Data Availability Statement in the manuscript PDF file). The data should be provided as part of the manuscript or its supporting information, or deposited to a public repository. For example, in addition to summary statistics, the data points behind means, medians and variance measures should be available. If there are restrictions on publicly sharing data—e.g. participant privacy or use of data from a third party—those must be specified.

Reviewer #1: Yes

**Answer:** Thank you for your positive feedback.

Reviewer #2: Yes

**Answer:** Thank you for your positive feedback.

4. Is the manuscript presented in an intelligible fashion and written in standard English?

PLOS ONE does not copyedit accepted manuscripts, so the language in submitted articles must be clear, correct, and unambiguous. Any typographical or grammatical errors should be corrected at revision, so please note any specific errors here.

Reviewer #1: Yes

**Answer:** Thank you for your positive feedback.

Reviewer #2: Yes

**Answer:** Thank you for your positive feedback.

5. Review Comments to the Author

Please use the space provided to explain your answers to the questions above. You may also include additional comments for the author, including concerns about dual publication, research ethics, or publication ethics. (Please upload your review as an attachment if it exceeds 20,000 characters)

**Answer:** Thank you for your thorough review and comments.

We have carefully considered your feedback and made the necessary revisions to address the issues raised. We sincerely appreciate your attention to detail and guidance in improving the manuscript. If you have any further concerns or additional comments, please do not hesitate to let us know.

Reviewer #1: This is an interesting paper and the content is very good but I suggest the paper can be improved in the following ways: Abstract
-Please correct all parts of the article according to the guidelines of the journal authors guideline In the methods section please bring year of performing of this study, sampling methods and data analysis methods -In the conclusion part, it is necessary to specify the researcher's proposal to improve the conditions and use of the beneficiaries

**Answer:** Thank you for your valuable feedback.

We have revised all parts of the article according to the guidelines of the journal authors guideline. Additionally, we have supplemented the year of performing of this study, sampling methods and data analysis methods of this study in the section of methods. In the conclusion part, the specific suggestions made by the researchers are also added.

Introduction
Please bring the following items 1- Definition of the research problem 2- The magnitude and importance of the study variable 3- Expressing the necessity of conducting the study Finally, the practical purpose of the study should be stated.

**Answer:** Thank you for your valuable insights regarding our manuscript.

1-According to your suggestion, we have reorganized the logic of the introduction and clarified our research question. There are two main research questions, which are the status quo of psychology resilience of foreign aid medical team members and the factors affecting their psychology resilience.

2-We added the magnitude and importance of study variables as you suggested, such as psychology resilience, self-efficacy, social support, etc.

3- We have explained the practical purpose of the study at the end.

This study aims to reveal the level of psychological resilience of this population and its related factors, with the goal of providing theoretical basis for effectively improving the psychological status of CMATMs and reducing the risk of mental diseases. Through the comprehensive analysis of these influencing factors, not only can provide a more accurate assessment of the psychological resilience of CMATMs, but also provide a scientific basis for follow-up intervention and support. Finally, it will help improve the overall adaptability and work stability of CMATMs, ensure the smooth development of foreign medical aid work, promote international medical cooperation and exchanges, enhance the friendship and mutual trust between different countries.

Methods
Please report the details scoring and validations of study tools

**Answer:** Thank you for your valuable feedback.

We have reported on the details of the study tools in section 2.2.2-2.2.5, such as scoring criteria and Cronbach's alpha coefficient.

Discussion
In the discussion section, it is necessary to compare the main results of the study with the results of other studies in this field. To strengthen the article, especially in the introduction and discussion section the following studies are suggested, please used and add to this manuscript references.
-Crowd Simulations and Determining the Critical Density Point of Emergency Situations
-Heat waves and adaptation: A global systematic review
_Medical tourism development: A systematic review of economic aspects
- Investigating the burden of disease dimensions (time-dependent, developmental, physical, social and emotional) among family caregivers with COVID-19 patients

**Answer:** Thank you for your careful feedback.

We have compared the main results of the study with the results of other studies in this field. Additionally, We have added a large number of references in accordance with your comments, respectively in reference 11, reference 13,reference 31, and reference 34.

Conclusion − What are your suggestion for future studies? Best regards

**Answer:** Thank you for raising this point.

We have added our suggestion for future studies in the conclusion section.

Multi-center study with large sample studies are needed to investigate the detailed factors affecting psychological resilience and explore the sociocultural adaptation of CMATMs in different countries in the future.

Reviewer #2: Dear authors
Thank you for your efforts to do this research. Below are some comments and suggestions to help improve the clarity and impact of your study.

Abstract:
Please specify the study duration.

**Answer:** Thank you for your suggestion.

We have added the specific study duration in the methods. This study was conducted from 3rd December, 2024 to 25th December, 2024.

The abstract provides a concise summary, but it lacks clarity in presenting the study's implications. Including a brief statement on how the findings can inform interventions would strengthen its impact.

**Answer:** Thank you for your valuable feedback.

We added a brief statement on how the findings can inform interventions would strengthen its impact. This study provides a theoretical basis for formulating psychological intervention measures according to the factors that affect the psychological resilience of CMATMs to help them establish a more adaptive defense mechanism and maintain good psychological resilience during foreign aid.

The conclusion section in the abstract could be more actionable, specifying the next steps or direct applications of the findings.
**Answer:** Thank you for your thoughtful comments.

We have revised our conclusion section to specify the next steps or direct applications of the findings.
Policymakers or healthcare administrators should regular assess CMATMs ' mental health status, strengthen mental health training, mobilize family and social support networks, improve infrastructure and welfare security policies.

Introduction section:
The transition to psychological resilience as a focus needs smoother integration. Consider elaborating on why psychological resilience, specifically, was prioritized over other psychological constructs.

**Answer:** Thank you for your insightful feedback.

We have revised the introduction to better integrate the focus on psychological resilience. In this revision, we emphasize the importance of resilience in the context of the challenges faced by foreign medical aid teams. These teams often encounter a wide range of psychological issues, and one of the key strategies for effectively coping with stress and maintaining mental health is resilience. This adjustment aims to provide a smoother transition and a clearer rationale for prioritizing psychological resilience in our study. We appreciate your suggestion, which has helped to improve the manuscript's coherence and focus.

References are well-cited but could benefit from the inclusion of more recent or diverse studies for broader relevance. For example, the authors can refer to the following articles on this topic:
https://www.mdpi.com/1660-4601/18/23/12548
https://www.frontiersin.org/journals/public-health/articles/10.3389/fpubh.2022.1034624/full
**Answer:** Thank you for your valuable suggestion.

We have carefully read and analyzed the references you have provided. We also have already included recommended references in the revised manuscript and cited them as reference 12 and reference 33 to enhancing the quality of our manuscript.

Methods:
The inclusion criteria are clear, but more details about the rationale for participant selection would be beneficial. Why was the range of roles (e.g., cooks and translators) included, and how do these roles contribute to the overall findings?

**Answer:** Thank you for your valuable feedback.

We have added the reasons for selecting non-medical members to the inclusion criteria, and in order to harmonize terminology, we will include medical and non-medical members in the study. While medical professionals were the core of CMATMs, the non-medical members were often an important support force of the team, which had an indirect impact on the emotional and mental health of the medical professionals. In order to fully understand the psychological resilience of the entire team, our study was not limited to the core medical staff, but also included non-medical staff.

The sample size (n=89) is relatively small. The limitations section addresses this, but a justification earlier in the methods could preempt concerns.

**Answer:** Thank you for your valuable insights regarding our manuscript.

We have added a justification for the sample size in Section 2.1 Study Design and participants, where we explain the sample size calculation method and provide reasons for the relatively small sample size. Due to the special research population of CMATMs, the number was relatively small. In addition, CMATMs were distributed in different countries and regions, it was difficult for researchers to contact all potential participants. Therefore, we did our best to select only 89 subjects. Relatively small sample size was undeniably a limitation of our research. This additional explanation aims to preempt concerns and clarify the rationale behind the chosen sample size.

Please explain the sampling method and how the sample size was estimated.

**Answer:** Thank you for your valuable feedback.

We have added the sampling method and the sample size calculation method in Section 2.1 Study Design and participants.

The use of validated scales like CD-RISC, GSES, SAS, and SDS is commendable. However:
Provide reliability coefficients (e.g., Cronbach's alpha) for these scales in the study sample, not just general references. Clarify if the scales were culturally adapted for Chinese participants beyond translation (e.g., if factor structures were re-validated).

**Answer:** Thank you for your attention to these details.

We have added the reliability coefficients in section 2.2.2-2.2.5, including Cronbach's alpha, for the scales used in our study. These coefficients are based on the data from our study sample, not just general references. Additionally, we have clarified that the scales were culturally adapted and re-validated for use with Chinese participants to enhance the transparency and rigor of our study.

Statistical methods are appropriate but require clarification: Explain why certain tests (e.g., ANOVA vs. Kruskal-Wallis) were chosen for specific analyses.

Include effect sizes alongside p-values to provide a more robust interpretation of significant results.

**Answer:** Thank you for your thoughtful suggestion.

We have added further clarification in Section 2.4 regarding the rationale for selecting the statistical methods used in our study. Additionally, we have included effect sizes alongside p-values to offer a more comprehensive interpretation of the significant results. These revisions aim to improve the clarity and robustness of the statistical analysis.

In order to study the influence of demographic variables on psychological resilience, T-test ANOVA and Kruskal-Wallis test were used. Depending on the normality of the data, when comparing 3 or more independent groups, ANOVA were used for data with normal distribution and homogeneity of variance. Otherwise, Kruskal-Wallis test was used. To investigate the relationship among psychological resilience, self-efficacy, anxiety and depression, Pearson correlation coefficient test was adopted and to predict the effects of demographic variables, self-efficacy, anxiety and depression on resilience, multiple linear regression analysis was used. The significance level for all variables was considered to be 0.05.

Results:
Ensure all abbreviations used in tables are defined in the captions or footnotes.

**Answer:** Thank you for your reminder.

We have revised all abbreviations used in the tables to enhance the clarity and accessibility of the manuscript for readers.

Highlight key findings directly in the text rather than leaving them for the reader to infer from the tables.

**Answer:** Thank you for your suggestion.

We have revised the manuscript to highlight the key findings directly in the text to improve the readability and ensure that the main outcomes are clearly emphasized for the reader.

The reported correlations between psychological resilience and variables like self-efficacy, anxiety, and depression are meaningful. However, the strength of these relationships should be contextualized within existing literature.

**Answer:** Thank you for your valuable feedback.

We have expanded the discussion section to provide context for the reported correlations between psychological resilience and variables like self-efficacy, anxiety, and depression. Specifically, we have referenced existing literature to help contextualize the strength of these relationships and provide a deeper understanding of their significance.

Multivariate Analysis: While the regression model is insightful, assumptions underlying the regression (e.g., multicollinearity, normality of residuals) are not addressed. Adding these details would strengthen the validity of the findings.

**Answer:** Thank you for your valuable feedback.

We have assessed multicollinearity using Variance Inflation Factors (VIF) and ensured that no significant issues were found. The test results are supplemented in Section 3.6 and Figure 6. Additionally, we have tested the normality of residuals using the residual test and visualized it with histogram and P-P plot. These checks confirm that the assumptions for the regression model are adequately met, further validating the robustness of our findings. We enclose the normality of residuals test results below for your review, which we also describe in Section 3.6.

Discussion Section:
The negative correlation between direct participation in public health emergencies and psychological resilience deserves deeper exploration. Was this due to trauma exposure, workload, or other factors?

**Answer:** Thank you for your valuable feedback.

We have expanded on the exploration of the negative correlation between direct participation in public health emergencies and psychological resilience. In our revision, we have discussed public emergencies are often accompanied by high stress and a large number of traumatic scenes. CMATMs face life-and-death decisions, patient suffering and threats to their own safety for long periods of time, which can trigger acute stress reactions or post-traumatic stress disorder (PTSD). Repeated exposure to other people's traumatic experiences can also lead to vicarious trauma, causing emotional numbness, anxiety or depression, and weakening psychological resilience. In addition, we also provided a number of interventions to enrich our discussions.

Suggestions for interventions, such as mindfulness strategies, are useful but could be made more specific and actionable.

**Answer:** Thank you for your insightful suggestion.

We have already incorporated specific and actionable mindfulness interventions into the revised manuscript. Additionally, we have revisited the discussion section and provided a more detailed description of the proposed intervention measures to enhance the clarity and applicability of the interventions.

The limitations are honestly presented but could be expanded to discuss potential response biases (e.g., social desirability bias in self-reported measures).

**Answer:** Thank you for your valuable comments.

We have revised the limitations based on your suggestions

We have expanded the limitations section according to your feedback. Our scales are self-report scales, which can lead to potential response bias, such as social desirability bias in self-reported measures. In the future, peer-report scales can be further developed to evaluate the psychological state of CMATMs.

I suggest enriching the discussion by addressing related studies. For example, I recommend the following article:
<https://journals.lww.com/jnmr/fulltext/2024/29030/relationship_between_resilience_and_social_and.12.aspx?context=latestarticles>

**Answer:** Thank you for your attention to related studies.

We have already included the recommended article in the revised manuscript and cited it as reference 39 to enhancing the depth of the discussion.

Conclusion:
The conclusion reiterates key findings but lacks actionable recommendations for policymakers or healthcare administrators. Consider framing specific strategies based on identified factors (e.g., training modules, resilience workshops).

**Answer:** Thank you for your valuable feedback.

We have revised the conclusion to include actionable recommendations for policymakers and healthcare administrators. Specifically, we have proposed strategies such as designing resilience assessment programs and workshops, strengthening mental health training, mobilizing family and social support networks, improving infrastructure and welfare security policies, which are based on the identified factors.

References:
The references are current and relevant, but ensure consistency in formatting (e.g., capitalization, use of journal abbreviations).

**Answer:** Thank you for your insightful feedback.

We have revised the reference format in accordance with the journal guideline to maintain consistency in formatting. We appreciate your attention to these details and have made the necessary adjustments to enhance the manuscript's overall quality.

Ethical Considerations:
Ethical approval and participant consent are adequately described. Consider briefly addressing how data security and participant anonymity were ensured during online questionnaire administration.

**Answer:** Thank you for your constructive feedback.

We followed your suggestions to add how data security and participant anonymity were ensured during online questionnaire administration in Ethical Considerations. In our study, randomly generated numbers were used to anonymously tag participants and only authorized staff were allowed to view or process the data to improve data security and participant anonymity during online questionnaire administration.

Language and Grammar:

The manuscript is generally well-written but has minor grammatical issues. For instance, "strong sense of loneliness" could be revised to "pronounced sense of loneliness" for precision.

**Answer:** Thank you for your valuable suggestion.

We have revised "strong sense of loneliness" to "pronounced sense of loneliness" for precision. Additionally, we have thoroughly checked the manuscript for other grammatical issues and made the necessary corrections. We appreciate your attention to detail and your valuable input.

In line 83, replace "are" with "were."

**Answer:** Thank you for your reminder.

We have replaced "are" with "were" in the line 115 in the revised manuscript.

6. PLOS authors have the option to publish the peer review history of their article ([what does this mean?](https://track.editorialmanager.com/CL0/https:%2F%2Fjournals.plos.org%2Fplosone%2Fs%2Feditorial-and-peer-review-process%23loc-peer-review-history/1/010f01955b273b4b-cb50b722-1bb9-4e12-930b-64ec03c1f07d-000000/ido72A3AIvhKnmZyRLkPa_wI3kGOQLuAeAdsD07CaBM=200)). If published, this will include your full peer review and any attached files.


If you choose “no”, your identity will remain anonymous but your review may still be made public.


**Do you want your identity to be public for this peer review?** For information about this choice, including consent withdrawal, please see our [Privacy Policy](https://track.editorialmanager.com/CL0/https:%2F%2Fwww.plos.org%2Fprivacy-policy/1/010f01955b273b4b-cb50b722-1bb9-4e12-930b-64ec03c1f07d-000000/-ciV4iriWO9z5tlLs3XkycKjOaKfrHCV6_ZUPYyLs1o=200).

Reviewer #1: No

Reviewer #2: No
