# Supplementary material for: Status and associated factors of psychological resilience of Chinese medical aid team members under public health emergencies in a cross-sectional study
Source: PLoS One. 2025 Jun 17;20(6):e0324952. doi: 10.1371/journal.pone.0324952 (PMC12173397; doi:10.1371/journal.pone.0324952)
Supplement: Attachment — Submitted filename: Response_to_Reviewers_auresp_2.docx [file pone.0324952.s004.docx]

**PONE-D-25-03434**

**Status and Associated Factors of Psychological Resilience of Chinese Medical Aid Team Members under Public Health Emergencies: A Cross-Sectional Study**

**PLOS ONE**

Dear Editor-in-Chief,

Thank you very much for your careful review and constructive suggestions with regard to our manuscript **“Status and Associated Factors of Psychological Resilience of Chinese Medical Aid Team Members under Public Health Emergencies: A Cross-Sectional Study”.**

We have carefully evaluated all the comments and suggestions, and responded to them in our revised manuscript accordingly. All the authors agreed upon and approved the contents of the manuscript. Again, we appreciate the scholarly work by the Editors/Reviewers, and hope our detailed revisions have addressed all the criticisms raised by the reviewers.

Please contact us with any further questions and we are looking forward to your positive consideration.

Sincerely,

Yinhua Liang

Teaching and Research Section of Clinical Nursing

Xiangya Hospital

Central South University
Changsha, 410008, China

liangyh@csu.edu.cn

Please include the following items when submitting your revised manuscript:

- A rebuttal letter that responds to each point raised by the academic editor and reviewer(s). You should upload this letter as a separate file labeled 'Response to Reviewers'.

**Answer:** Thank you for your suggestion.

We have included a separate **"Response to Reviewers"** document addressing each point raised.

- A marked-up copy of your manuscript that highlights changes made to the original version. You should upload this as a separate file labeled 'Revised Manuscript with Track Changes'.

**Answer:** Thank you for your reminder.

We have provided a marked-up copy of the manuscript labeled **'Revised Manuscript with Track Changes'** for your review.

- An unmarked version of your revised paper without tracked changes. You should upload this as a separate file labeled 'Manuscript'.

**Answer:** Thank you for your reminder.

We have provided an unmarked version of the revised paper labeled **'Manuscript'** for your review.

If you would like to make changes to your financial disclosure, please include your updated statement in your cover letter. Guidelines for resubmitting your figure files are available below the reviewer comments at the end of this letter.
**Answer:** Thank you for your notice.

We do not need to make any changes to our financial disclosure. In addition, we have followed the guidelines provided for resubmitting the figure files.

If applicable, we recommend that you deposit your laboratory protocols in protocols.io to enhance the reproducibility of your results. Protocols.io assigns your protocol its own identifier (DOI) so that it can be cited independently in the future. For instructions see: <https://journals.plos.org/plosone/s/submission-guidelines#loc-laboratory-protocols>. Additionally, PLOS ONE offers an option for publishing peer-reviewed Lab Protocol articles, which describe protocols hosted on protocols.io. Read more information on sharing protocols at <https://plos.org/protocols?utm_medium=editorial-email&utm_source=authorletters&utm_campaign=protocols>.
**Answer:** Thank you for the thoughtful suggestion.

Our team carefully considered your recommendation. However, as we have provided a detailed description of our methods in the manuscript, we are not planning to deposit our laboratory protocols in protocols.io at this time. Nonetheless, we sincerely appreciate the suggestion and will keep it in mind for future projects.

We look forward to receiving your revised manuscript.

Journal Requirements:

Please review your reference list to ensure that it is complete and correct. If you have cited papers that have been retracted, please include the rationale for doing so in the manuscript text, or remove these references and replace them with relevant current references. Any changes to the reference list should be mentioned in the rebuttal letter that accompanies your revised manuscript. If you need to cite a retracted article, indicate the article’s retracted status in the References list and also include a citation and full reference for the retraction notice.
**Answer:** Thank you for your valuable guidance.

We have carefully reviewed the reference list to ensure its completeness and accuracy. We confirm that all cited papers are, to the best of our knowledge, valid and have not been retracted. We have not knowingly cited any retracted papers in our manuscript.

Additional Editor Comments :

Dear Authors,

Thank you for your revised manuscript and comprehensive responses to the reviewers’ comments. You have addressed many important points with clarity and care. In addition to the reviewers’ comments, please consider the following detailed suggestions to further improve the quality, clarity, and impact of your work:
**Answer:** Thank you very much for considering our work and for your valuable guidance.

We have revised our manuscript one by one according to all reviewers' comments and provided a peer-to-peer response. Additionally, we have made all modifications in blue color. We are looking forward to your positive consideration.

1. Title and Abstract
Title: The title is clear and descriptive. You may consider adding “in a cross-sectional study” at the end for greater methodological clarity.
**Answer:** Thank you for your suggestion.

As suggested, we have revised the title to: “Status and Associated Factors of Psychological Resilience of Chinese Medical Aid Team Members under Public Health Emergencies in A Cross-sectional Study”.

Abstract:

You’ve added important methodological details. Still, the conclusion should present more direct applications. For instance, “help them establish a more adaptive defense mechanism” could be made more actionable by suggesting how or through what mechanism (e.g., targeted interventions, training programs).
**Answer:** Thank you for your insightful comments.

We have revised the conclusion to include more concrete recommendations based on our results. We now propose that interventions could include: regular assessment of CMATMs’ mental health status, strengthening mental health training programs, mobilizing family and social support networks, and improving infrastructure and welfare security policies.

Reduce redundancy in wording and ensure all key findings are reported with clarity and brevity.
**Answer:** Thank you for valuable feedback.

We have removed unnecessary phrases and rephrased sentences to be more concise. We have also ensured that the reporting of our key findings is direct and to the point, avoiding any ambiguity.

2. Introduction
The revised introduction has improved coherence. However:

Clarify why psychological resilience was prioritized over other constructs such as coping or burnout—e.g., resilience may offer a broader protective framework with intervention potential.
**Answer:** Thank you for your feedback on the introduction.

We have expanded the introduction to explicitly address this point. We now emphasize that psychological resilience provides a broader, more comprehensive framework for understanding and promoting positive adaptation in the face of adversity. Resilience focuses on how individuals actively adapt to adversity and even grow from the experience, offering a more holistic perspective on mental well-being that goes beyond merely mitigating the negative impacts of stress.

Furthermore, we highlight that focusing on resilience can help us understand the psychological adaptation process of medical aid workers more comprehensively and offer a broader protective framework with intervention potential. We believe this is particularly relevant for medical aid team members who face a wide range of challenges and require a proactive approach to maintaining their psychological health.

In lines 85–87, when stating the lack of studies on CMATMs’ resilience, consider framing this as a critical research gap given their frontline role in China's foreign health aid policy.
**Answer:** Thank you for highlighting the importance of emphasizing the research gap.

We have revised lines 85-87 to explicitly state this critical research gap. We now emphasize that the limited research on CMATMs’ psychological resilience represents a significant oversight, considering their pivotal role in implementing China’s foreign health aid policy and their exposure to unique stressors in challenging environments. By highlighting this gap, we aim to underscore the urgency and relevance of our study in addressing the psychological well-being of these vital healthcare professionals.

Consider briefly mentioning a theoretical foundation (e.g., conservation of resources theory or stress-appraisal models) to ground your variable selection.
**Answer:** Thank you for your valuable feedback.

We have added a brief mention of the stress-coping theory to the introduction. We now explain that our study revealed the level of psychological resilience of CMATMs and its related factors based on the stress-coping theory.

3. Methods
Sample Size Justification: Clearly explain how the 35 explanatory variables were identified, and clarify how many were retained in regression to avoid overfitting concerns.
**Answer:** Thank you for your valuable feedback.

We have carefully reviewed our analysis and consulted with a statistical expert to address these points. We would also like to sincerely apologize for the previous misstatement regarding the number of explanatory variables. Upon closer review and verification, we confirm that there are 34, not 35, explanatory variables in our dataset. We regret any confusion this may have caused and appreciate the reviewer’s attention to detail.

Explanatory variables are also known as independent variables. For the sake of easier understanding, we uniformly refer to them as independent variables.

The 34 independent variables comprised 31 demographic characteristics (detailed in Section 2.2.1, ‘Demographic Information Questionnaire’ which includes two parts with 11 and 20 variables, respectively) and the total scores from three validated scales (Self-Efficacy Scale, Anxiety Scale, and Depression Scale). Furthermore, we also supplemented how variables were identified in Section 2.2.1, ‘Demographic Information Questionnaire’. According to the purpose of the study, research team reviewed previous relevant literature and in combination with the special working environment and living conditions of medical aid team members, formulated a demographic information questionnaire based on the stress-coping theory.

**31 demographic variables:** The first part included 11 variables, namely age, sex, marital status, highest level of education education, occupation, level of the dispatching organization, professional title, prior overseas experience before participating in foreign aid work, classification of hardship areas in recipient countries, family support, and family fertility.

The second part included 20 variables, namely satisfaction with family life, satisfaction with work, smoking status, frequency of drinking, loneliness, culture and entertainment, frequency of exercise, average daily Internet use time, economic subsidy policy, professional title promotion policy, material condition, political situation, social security situation, language adaptation situation and climate adaptation situation of the recipient country, history of mosquito or insect bites, history of typhoid or malaria or COVID-19 infection, and direct participation in public health emergency response missions in recipient countries.

The demographic variables with statistical significance in the univariate analysis and the total scores of the three scales were taken as independent variables for multiple linear regression. Ultimately, 14 demographic variables and the total scores of three scales were included in the regression analysis.

All the above revisions and clarifications have been supplemented in "2.1 Study Design and participants" and "2.2.1 Demographic information questionnaire".

Inclusion of Non-Medical Staff: Well-justified. Consider reporting the medical vs. non-medical distribution (e.g., n or %) clearly in the results section for transparency.
**Answer:** Thank you for the suggestion.

We have now added the distribution of medical and non-medical staff in the results section 3.1. The vast majority were medical staff (89.9%), and non-medical staff accounted for 10.1%.

Study Tools:

Cronbach’s alpha is reported well. Add one sentence clarifying whether any confirmatory factor analysis or cultural validation (beyond translation) was performed or cited.
**Answer:** Thank you for your valuable feedback.

We have added a sentence to the methods section to clarify the validation information for the four scales used in this study.

For example, The Chinese version of the CD-RISC had undergone cross-cultural debugging, confirmatory factor analysis and revision, modifying the five dimensions of the original scale to three dimensions. Reliability, structural validity and predictive validity tests were conducted in the context of Chinese culture to verify the validity and reliability of the Chinese version of GSES. For the SAS, through exploratory factor and confirmatory factor analysis, the factors presented in different populations were stable. SDS was proved to be applicable to the study of depression in Chinese population through the exploratory factor and confirmatory factor analysis

Statistical Analysis:

You explain the use of parametric vs. non-parametric tests well. Still, please report effect sizes (e.g., η², Cohen’s d, or r) with significance values in key results to improve interpretability.
**Answer:** Thank you for your valuable feedback.

As suggested, to evaluate the strength of the correlation, we calculated the Pearson correlation coefficient (*r*) as the effect size. we have now added *r* value in Section 2.4). The value range of *r* is from -1 to +1. The larger the absolute value, the stronger the correlation.

Additionally, in the results of multiple linear regression, we also reported the adjusted R².

4. Results
Define all abbreviations directly in each table’s footnotes, even if introduced earlier.
**Answer:** Thank you for your reminder.

We have revised all abbreviations directly in each table’s footnotes, even if introduced earlier.

Consider reordering variables in Tables 1 and 2 for better logical flow (e.g., demographics before contextual or behavioral factors).
**Answer:** Thank you for your suggestion.

We have reordered the variables in Tables 1 and 2 to present the demographic characteristics before the contextual or behavioral factors.

Highlight in the narrative how many participants fell into each resilience classification, as these are reported numerically in Table 5 but not elaborated upon in-text.
**Answer:** Thank you for pointing out this.

We have now added a sentence in the section “3.5 The influence of different degree of psychological status on psychological resilience” to explicitly state the number of participants falling into each resilience classification, as presented in Table 5.

The results showed that the proportions of those with excellent, good, average and poor psychological resilience were 30.3%, 23.6%, 25.8, 20.2, respectively.

For Table 6 (Multivariate Model), include the adjusted R² value to give readers a sense of overall model strength.
**Answer:** Thank you for your careful feedback.

We carefully considered your suggestion and, after further discussion, we believe that incorporating the adjusted R² value into the narrative of Section 3.6, rather than directly into Table 6, is a more appropriate approach in this context.

Therefore, we have added a sentence to Section 3.6 stating that “The adjusted R² for the multivariate model was 0.850, indicating that the model explained approximately 85.0% of the variation degree of psychological resilience, and the model had a relatively high goodness of fit.”

5. Discussion
The link between direct emergency response and reduced resilience is very important. Expand this further by discussing potential mechanisms like trauma exposure, decision fatigue, or moral injury.
**Answer:** Thank you for highlighting the importance.

We have expanded the discussion section to explore these potential mechanisms in more detail. Public emergencies are inherently characterized by high-stress environments compounded by a multitude of trauma scenes, including mass casualties, severe illness, and the suffering and death of patients. Team members directly involved in the response face life-and-death decisions, patient suffering and threats to their own safety for long periods of time, which can trigger acute stress reactions or post-traumatic stress disorder (PTSD). Repeated exposure to other people's traumatic experiences can also lead to vicarious trauma, causing emotional numbness, anxiety or depression, and weakening psychological resilience. Unlike routine medical practice, these situations often involve unpredictable circumstances, such as resource scarcity, ethical dilemmas surrounding patient prioritization, and the potential for exposure to highly contagious pathogens. Rescue workers need to make a large number of decisions in a short period of time, such as determining the priority order for treating the injured and choosing the best rescue plan, etc. Long-term and high-intensity decision-making may lead to the depletion of cognitive resources, reduce an individual's self-control ability and the ability to cope with stress, thereby affecting psychological resilience.

The association with professional title could be explained through concepts such as job autonomy, leadership buffering, or perceived control.
**Answer:** Thank you for your valuable feedback.

We have expanded the discussion section to incorporate these ideas. We now discuss how individuals with higher professional titles may experience greater job autonomy, allowing them more control over their work and reducing stress. We also explore the possibility that these individuals may benefit from leadership buffering, where supervisors provide support and protect them from excessive demands.

When discussing mindfulness interventions, support the suggestion by citing studies that have shown measurable impacts of such strategies on aid workers or healthcare professionals.
**Answer:** Thank you for your valuable comments.

We have now added citations to relevant studies that have shown measurable impacts of mindfulness interventions on aid workers or healthcare professionals in the discussion section. Specifically, we have cited studies that demonstrate the effectiveness of mindfulness-based interventions in reducing burnout and enhancing the resilience of healthcare professionals

Consider providing clearer links between findings and proposed interventions, e.g., how family support could be mobilized through organizational communication strategies or flexible leave policies.
**Answer:** Thank you for your valuable feedback.

We have expanded the discussion section to provide more specific examples of how the proposed interventions can be implemented to address the identified needs. For instance, we now stated that relevant institutions should establish smooth information channels, organize regular family symposiums, set up family support hotlines, provide psychological counseling and support services for team members and their families, eliminating their doubts and concerns. At the same time, the dispatch and rotation mechanism of foreign aid medical team members should be improved, and personalized dispatch plans can be provided for a period of time. For example, the family visit leave for team members can be appropriately increased, or they can be allowed to return to their home country for leave in advance after completing a certain amount of work.

The policy relevance of your findings could be strengthened by summarizing what institutions or agencies could do in practical terms (e.g., onboarding protocols, resilience screening, psychosocial briefings).
**Answer:** Thank you for your valuable feedback.

We have revised discussion section by summarizing what institutions or agencies could do in practical terms. We further expanded the discussion around aspects such as onboarding protocols, psychological condition screening and assessment.

6. Conclusion
The conclusion is now more structured. Consider presenting it in three parts:

A summary of the key findings;
**Answer:** Thank you for your valuable feedback.

We have added a summary of the key findings. This study revealed that CMATMs exhibited lower psychological resilience when characterized by a low sense of self-efficacy, a lower professional title, direct involvement in emergency public health rescue efforts, pronounced sense of loneliness, and limited access to cultural and recreational activities in the recipient area.

A clear set of implications for practice;
**Answer:** Thank you for your valuable feedback.

We have added a clear set of implications for practice. This study provides guidance for the relevant institutions to select appropriate intervention measures according to the influence factors, which can help foreign aid medical team members enhance their ability to cope with stress, maintain a good working state and efficiency, and thereby enhance the cohesion and collaboration ability of the foreign aid team.

A brief roadmap for future studies (e.g., “multi-country comparative studies using longitudinal methods”).
**Answer:** Thank you for your valuable suggestion.

We have added a brief section to the conclusion outlining potential avenues for future research. Multi-country comparative studies with large sample using longitudinal methods are needed to investigate the detailed factors affecting psychological resilience and explore the sociocultural adaptation of CMATMs in different countries in the future.

7. Ethical Considerations and Transparency
You provide good detail. Consider specifying whether the online platform used for data collection complied with national data privacy regulations (e.g., China’s Cybersecurity Law).
**Answer:** Thank you for your valuable feedback.

We have added that the online platform used for data collection complied with China’s Cybersecurity Law in section 2.5 “Ethical considerations”.

8. Language and Style
The manuscript is generally well-written, but consider a final grammar check focusing on:

Article use ("a large number of trauma scenes");
**Answer:** Thank you for your valuable feedback.

We re-examined the content and writing methods of the article. We have revised some sentences to "a large number of trauma scenes". Additionally, we have now engaged a native English speaker to proofread the manuscript. This included a review for grammar, spelling, punctuation, and overall clarity.

Verb tense consistency in results;
**Answer:** Thank you for your suggestions.

We have unified the tenses of the results section. In the result part, we uniformly used the past tense.

Concise language in long compound sentences;
**Answer:** Thank you for your suggestions.

We have concised language in long compound sentences.

Replacing “strong sense of loneliness” with “pronounced sense of loneliness” as previously suggested.
**Answer:** Thank you for your suggestions.

We have replaced “strong sense of loneliness” with “pronounced sense of loneliness”.

In several sections, repeated use of “CMATMs” could be replaced with “participants” or “team members” once the term is clear.
**Answer:** Thank you for pointing out this.

We have carefully reviewed the manuscript and replaced instances of repeated “CMATMs” with “participants” or “team members” where appropriate, ensuring clarity is maintained.

Final Recommendation
Your study addresses an important gap in global health resilience research. The manuscript is significantly improved and offers valuable insights. With the above refinements—especially in the discussion and stylistic clarity—it will be ready for publication.
**Answer:** Thank you very much for your positive and constructive feedback.

We are delighted to hear that you feel the manuscript has been significantly improved and offers valuable insights into global health resilience research. We have carefully addressed each of your remaining points, incorporating the recommended changes to further enhance the manuscript.

We are grateful for your guidance throughout this review process.

Reviewers' comments:

Reviewer's Responses to Questions

**Comments to the Author**

1. If the authors have adequately addressed your comments raised in a previous round of review and you feel that this manuscript is now acceptable for publication, you may indicate that here to bypass the “Comments to the Author” section, enter your conflict of interest statement in the “Confidential to Editor” section, and submit your "Accept" recommendation.

Reviewer #3: All comments have been addressed

**Answer:** Thank you for your valuable feedback.

We sincerely appreciate your attention to detail and guidance in improving the manuscript.

2. Is the manuscript technically sound, and do the data support the conclusions?

The manuscript must describe a technically sound piece of scientific research with data that supports the conclusions. Experiments must have been conducted rigorously, with appropriate controls, replication, and sample sizes. The conclusions must be drawn appropriately based on the data presented.

Reviewer #3: Yes

**Answer:** Thank you for your positive feedback.

3. Has the statistical analysis been performed appropriately and rigorously?

Reviewer #3: Yes

**Answer:** Thank you for your positive feedback.

4. Have the authors made all data underlying the findings in their manuscript fully available?

The [PLOS Data policy](http://www.plosone.org/static/policies.action#sharing) requires authors to make all data underlying the findings described in their manuscript fully available without restriction, with rare exception (please refer to the Data Availability Statement in the manuscript PDF file). The data should be provided as part of the manuscript or its supporting information, or deposited to a public repository. For example, in addition to summary statistics, the data points behind means, medians and variance measures should be available. If there are restrictions on publicly sharing data—e.g. participant privacy or use of data from a third party—those must be specified.

Reviewer #3: Yes

**Answer:** Thank you for your positive feedback.

5. Is the manuscript presented in an intelligible fashion and written in standard English?

PLOS ONE does not copyedit accepted manuscripts, so the language in submitted articles must be clear, correct, and unambiguous. Any typographical or grammatical errors should be corrected at revision, so please note any specific errors here.

Reviewer #3: Yes

**Answer:** Thank you for your positive feedback.

6. Review Comments to the Author

Please use the space provided to explain your answers to the questions above. You may also include additional comments for the author, including concerns about dual publication, research ethics, or publication ethics. (Please upload your review as an attachment if it exceeds 20,000 characters)

Reviewer #3: The manuscript was well written. All comment from the reviewers (from the previous round) had been addressed. Following are some additional feedback to further improve the manuscript:
**Answer:** Thank you very much for your positive feedback.

We have carefully considered each of your points and implemented the recommended changes. We believe these revisions have further strengthened the manuscript and enhanced its clarity, impact, and overall quality. We truly appreciate your expertise and guidance throughout this review process. Thank you again for your valuable contributions.

Methodology:
Please describe the study design of this study
Line 107: Please check the spelling for form

**Answer:** Thank you for your suggestion.

We have revised the spelling for form in line 116.

Line 187: Please provide the full name for the company for excel
**Answer:** Thank you for your suggestion.

We have updated the manuscript to include the full name of the company in line 208: Microsoft Excel 2019.

Results:
Please rename Table 5 to "The level of Self Efficacy, Anxiety, and Depression on Psychological Resilience
**Answer:** Thank you for your valuable feedback.

We have renamed Table 5 to "The level of Self Efficacy, Anxiety, and Depression on Psychological Resilience”.

Please do English proof reading to the manuscript.

**Answer:** Thank you for your feedback.

We have now engaged a native English speaker to proofread the manuscript. This included a review for grammar, spelling, punctuation, and overall clarity. We believe this has significantly improved the language quality of the manuscript and addressed any potential issues.

7. PLOS authors have the option to publish the peer review history of their article ([what does this mean?](https://journals.plos.org/plosone/s/editorial-and-peer-review-process#loc-peer-review-history)). If published, this will include your full peer review and any attached files.


If you choose “no”, your identity will remain anonymous but your review may still be made public.


**Do you want your identity to be public for this peer review?** For information about this choice, including consent withdrawal, please see our [Privacy Policy](https://www.plos.org/privacy-policy).

Reviewer #3: No
